# Outcomes of VDPACE with an immunomodulatory agent as a salvage therapy in relapsed/refractory multiple myeloma with extramedullary disease

**DOI:** 10.1002/jha2.275

**Published:** 2021-09-16

**Authors:** Al‐Ola Abdallah, Ghulam Rehman Mohyuddin, Nausheen Ahmed, Meera Mohan, Wei Cui, Leyla Shune, Zahra Mahmoudjafari, Joseph McGuirk, Siddhartha Ganguly, Shebli Atrash

**Affiliations:** ^1^ Division of Hematologic Malignancies & Cellular Therapeutics University of Kansas Medical Center Westwood Kansas USA; ^2^ University of Kansas Medical Center Kansas City Kansas USA; ^3^ Division of Hematology/Oncology Medical College of Wisconsin Milwaukee Wisconsin USA; ^4^ Division of Pathology & Laboratory Medicine University of Kansas Medical Center Kansas City Kansas USA; ^5^ Levine Cancer Institute Carolinas Healthcare System Charlotte North Carolina USA

**Keywords:** extramedullary disease, multiple myeloma, plasmacytoma, salvage therapy

## Abstract

Extramedullary disease (EMD) is an aggressive form of multiple myeloma (MM). Confirming the presence of plasma cells outside the bone marrow makes the diagnosis of EMD. There is no clear consensus on the management of EMD in MM, and this entity continues to remain an unmet need. Rapidly controlling EMD to prevent end‐organ damage is a priority. Retrospectively, we reviewed our database for patients with EMD that received treatment with bortezomib, dexamethasone, cisplatin, doxorubicin, cyclophosphamide, etoposide (VDPACE) plus an immune modulator (IMiD) regimen. We identified 21 patients with a median age of 61 years. Ten patients received a VDPACE based regimen as a bridge to autologus stem cell transplant (ASCT). After a median follow‐up of 51.4 months, the median overall survival (OS) and progression‐free survival were 14.9 months (95% CI: 7.8‐NA) and 5.5 months (95% CI: 3.9‐NA), respectively. The overall response rate was 76%, with a manageable safety profile. Interestingly, these results were similar regardless of the presence of high‐risk cytogenetics. The safety profile was acceptable. In conclusion, a salvage VDPACE‐based regimen plus an IMiD remains an effective and safe bridging therapy to future ASCT and immunotherapy in relapsed/refractory multiple myeloma patients with EMD.

## 1 INTRODUCTION

Multiple myeloma (MM) is characterized by a proliferation of malignant plasma cells with strong dependence on the bone marrow microenvironment [1]. Despite novel agents and increased better outcomes over the past years, MM is still an incurable disease marked by a relapse‐remission pattern [2]. Extramedullary disease (EMD) is an aggressive presentation both at diagnosis and relapse [3]. EMD is defined by the presence of soft tissue plasmacytomas or plasma cell infiltration at various sites outside of the bone marrow (mainly liver, skin, central nervous system (CNS), pleural effusion, kidneys, lymph nodes, and pancreas) [4, 5]. The diagnosis of EMD is typically made by the presence of pathologic soft tissue masses by physical examination or imaging [computed tomography (CT) scan, 18‐fluoro‐deoxyglucose positron emission tomography‐computed tomography (18F‐FDG PET), magnetic resonance imaging, or ultrasound], and confirmed by a biopsy [6].

The reported incidence of EMD in newly diagnosed MM (NDMM) ranges from 7% to 18%. Also, 6–20% of patients develop EMD later in relapsed/refractory multiple myeloma (RRMM) with EMD [7, 8, 9, 10]. EMD is associated with an adverse prognosis in both NDMM and RRMM patients and tends to be resistant to proteasome inhibitors (PI) and immunomodulatory agents (IMiD) [4, 11]. High‐risk cytogenetics was reported in RRMM with EMD [12, 13, 14]. The mechanisms of extramedullary spread in MM are unclear. Some studies suggest decreased expression of adhesion molecules (VLA‐4 and CD‐44), loss of CD56, downregulation of P‐selectin, low expression of chemokine receptors, downregulation of CXCR4, high frequency of RAS mutations, or increase angiogenesis are possible mechanisms in EMD [1, 5, 15]. Optimal management of EMD is unknown, although several prospective studies reported EMD; however, none of them established a solid approach to treating RRMM with EMD [16, 17, 18, 19, 20]. Aggressive RRMM with EMD needs rapid cytoreduction for disease control, which may not be possible with standard novel therapies. This study aimed to analyze the clinical outcomes of the application of salvage VDPACE regimen with an IMiD in patients with aggressive RRMM with EMD.

## 2 PATIENTS AND METHODS

A retrospective analysis was performed from January 2012 to December 2020 for patients with RRMM with EMD (identified based on the International Myeloma Working Group (IMWG) criteria [21] who received salvage therapy VDPACE + IMiD at the University of Kansas Health System. The diagnosis of EMD was based on the histology of tumor bulk or, if a biopsy was not possible, on imaging using CT, diffusion‐weighted magnetic resonance imaging, or 18F‐FDG PET. A full listing of inclusion and exclusion criteria are addressed in Table 1

**TABLE 1:** Inclusion and exclusion criteria

**Inclusion criteria** Patients must meet all of the following criteria to receive VDPACE+ IMiD per the institutional protocol: Written informed consent in accordance with institutional guidelines Confirmed diagnosis of disease progression multiple myeloma Eastern Cooperative Oncology Group (ECOG) performance status of 0 or 1 Adequate hepatic function with total bilirubin < 2× upper limit of normal (except Gilbert's syndrome), AST < 2.5 × ULN and ALT < 2.5 × ULN Adequate renal function with CrCl > 30 mL/min, calculated using the formula of Cockroft Adequate hematopoietic function: total WBC > 1500/mm^3^, ANC > 1000/mm^3^, and platelet count > 50,000/mm^3^ (in whom < 50% of bone marrow nucleated cells are plasma cells) or > 20,000/mm^3^ (in whom > 50% of bone marrow nucleated cells are plasma cells)	
**Exclusion Criteria** Patients meeting any of the following exclusion criteria are not eligible to receive VDPACE +IMiD per the protocol: Unstable cardiovascular function: symptomatic ischemia, congestive heart failure of NYH Class III/IV or EF per echocardiogram < 45%, myocardial infarction within 3 months and uncontrolled significant conduction abnormalities Active infection requiring antibiotics, antiviral or antifungals within one week prior to first dose Active hepatitis A, B, or C infection Uncontrolled GI symptoms (diarrhea, nausea or vomiting) Serious psychiatric conditions Lack of social support and caregiver	

Our database identified 21 patients. Descriptive statistics were utilized in data analysis for patient characteristics, disease course, and outcomes. Survival analysis using the Kaplan Meier method was done using the software R (Vienna, Austria) v2.15.1 and survival package [22, 23, 24]. Responses were evaluated using the IMWG criteria [25].

## 3 TREATMENT PROTOCOL

The regimen VDPACE + IMiD consisted of high‐dose dexamethasone (40 mg on days 1–4) and a 4‐day continuous infusion of cisplatin 10 mg/m^2^, cyclophosphamide 40 mg/m^2^, etoposide 40 mg/m^2^, doxorubicin 10 mg/m^2^, and bortezomib 1 mg/m^2^ on days 1, 4, 8, and 11 in addition to an IMiD (thalidomide or lenalidomide) administered every 4 to 6 weeks (Table 2) [26, 27]. All patients received lenalidomide except two patient who received thalidomide due to cytopenia. The doses of individual drugs were modified according to standard practice whenever necessary (the main cause of dose adjustment in this study was renal failure). An initial infusion of cisplatin, cyclophosphamide, etoposide, and doxorubicin was provided on an inpatient basis on days 1–4; subsequent therapy was delivered on an outpatient basis if the patient's condition was deemed stable enough. All patients received infusions via a central venous catheter. All patients received supportive therapy, including oral fluconazole, oral levofloxacin, oral cotrimoxazole (single strength). Also, patients received oral acyclovir prophylactically during neutropenia, and they continued until the absolute neutrophil count (ANC) was ≥1.0  × 10^9^/L for two consecutive days. Proton pump inhibitor, subcutaneous PEGylated filgrastim (between day seven till recovery), and daily subcutaneous prophylactic‐dose low‐molecular‐weight heparin were given. Patients received packed red blood cells and pooled platelet transfusions according to prevailing guidelines. Clinicians assessed responses after the first therapy cycle; cycles could be repeated (in 4–6 weeks) after the recovery of ANC to >1 × 10^9^/L and platelet count to >100 × 10^9^/L. Each treating physician decided on the number of cycles of therapy based on response and tolerability. Patients who were discharged from the hospital were required to follow up with daily laboratory testing to spot the potential for the need for platelet and/or red blood cell transfusion, with at least a weekly in‐person office visit with a provider until laboratory values fell into the normal range.

**TABLE 2:** Description of chemotherapy protocol

Treatment agents	Variable dosing/day	Route	Schedule
Bortezomib	1.0 mg/m^2^	Subcutaneous	Days 1,4,8 and 11
Dexamethasone	20–40 mg^a^	Oral	Day 1–4
Cisplatin	7.5‐10 mg/m^2^ ^a^	IV continuous infusion	Day 1–4
Doxorubicin	7.5‐10 mg/m^2^ ^a^	IV continuous infusion	Day 1–4
Cyclophosphamide	300–400 mg/m^2^ ^a^	IV continuous infusion	Day 1–4
Etoposide	30–40 mg/m^2^ ^a^	IV continuous infusion	Day 1–4
Immunomodulator agents^b^			
Thalidomide	100–200 mg^a^	Oral	Day 1–14^c^
Lenalidomide	15–25 mg^a^	Oral	Day 1–14^c^
Supportive care agents			
Pegfilgrastim	6 mg	Subcutaneous	Day 7
Fosaprepitant	150 mg	Intravenous	Day 1
Ondansetron	16 mg	Oral	Day 1–5
Methotrexate ^d^	6 mg/5 mL	Intrathecal	Days 1 and 8
Cytarabine ^d^	100 mg/5 mL	Intrathecal	Days 4 and 11

^a^ Dose adjusted based on performance status, comorbidities. Dose adjustment of 25% reduction was made for those with PS of 2, age >75 years, and comorbidities. For those with CrCl < 30 mL/min Cisplatin was omitted.

^b^ Immunomodulator drug were used was either thalidomide or lenalidomide.

^c^ Treatment was discontinued if platelets is less than 25,000.

^d^ Intrathecal chemotherapy (methotrexate and cytarabine) was administered in those with CNS involvement. Platelets counts should be more than 50,000.

## 4 RESULTS

### 4.1 Patients characteristics

We identified 21 patients with extramedullary RRMM treated with VDPACE + IMiD. The median age at the start of VD‐PACE + IMiD regimen for EMD was 61 (range 41–77) years. At the initial diagnosis of MM, primary EMD was present in four (19%).

At presentation, 11 patients (52%) had high‐risk cytogenetics [17p deletions, 1q21 gains, 1p deletion, *t* (4;14), *t* (14;16), and *t* (14;20)] by bone marrow biopsy. Muscle, skin, and soft tissue manifestation were the most frequent EMD presentation. CNS involvement was seen in nine patients (43%). Those patients who were confirmed with CNS myeloma via analysis cerebrospinal fluid (CSF) studies that confirmed elevated monoclonal plasma cells, these patients received intrathecal chemotherapy (methotrexate and/or cytarabine) twice a week, with two consecutive CSF analysis confirmed clearance of plasma cells the intrathecal chemotherapy (IT) chemotherapy was discontinued.

In our cohort, patients had been treated with a median of three (range 1–8) prior lines of therapy. Twelve patients (57%) had IgG isotype, and seven patients (33%) had international staging system (ISS) stage III disease at diagnosis. All patients were exposed to PI (bortezomib/carfilzomib). Lenalidomide, pomalidomide, and thalidomide had been previously given in 18 (86%) patients. Six (29%) patients were previously treated with daratumumab. Eighteen (86%) patients had previously undergone autologous stem cell transplants (SCT). All patients were refractory to the last line of therapy. Table 3 summarizes patients' characteristics.

### 4.2 Responses

The overall response rate (ORR) was 76%: complete response (CR) in 10% of patients, very good partial response (VGPR) in 43% of patients, partial response (PR) in 33% of patients. Also, 10% of patients reported a stable disease. The median time to response was 21 days (14–45). For those who were double refractory to PI+ IMiD (*n* = 16), the ORR was 75%. Among the 16 patients who had a response, nine patients proceeded to ASCT and/or allogeneic stem cell transplant (Allo‐SCT). Seven patients did not proceed to ASCT, despite having a response to the VDPACE+ IMiD regimen. Among these seven patients who did not to pursue ASCT, they used different regimens as the following: two patients received daratumumab/pomalidomide/dexamethasone, one patient received carfilzomib/pomalidomide/dexamethasone, one patient received carfilzomib/cyclophosphamide/dexamethasone, one patient proceeds to enroll in a clinical trial, one patient decided not to pursue any treatment and received palliative radiation, and one patient had progression prior evaluation for ASCT due to delay to evaluate this patient and was reinduce with a different regimen. Table 4 summarizes the response rates.

**TABLE 3:** Characteristics of patients with RRMM (*n* = 21)

Characteristics	Rates
Gender, male: female	11:10
Age, years, median (range)	61 (41–77)
Race, number of patients (%)	
Caucasian	12 (57%)
African American	7 (33%)
Asian	1 (5%)
Hispanic	1 (5%)
Multiple myeloma paraprotein, number of patients (%)	
IgG	12 (57%)
Non‐IgG	8 (38%)
Light chain	1 (5 %)
Baseline ISS stage, number of patients (%)	
Stage III	7 (33%)
Stage II	7 (33%)
Stage I	5 (24%)
Unknown	2 (10%)
Cytogenetics, number of patients (%)	
High risk	11 (52%)
Standard risk	8 (38%)
Unknown	2 (10%)
Median number of previous lines of therapy for relapsed/refractory myeloma (range)	3 (1–8)
Received PI	21 (100%)
Refractory to PI	18 (86%)
Received IMiD	18 (86%)
Refractory to IMiD	17 (81%)
Refractory to Daratumumab	6 (29%)
Double refractory (PI and IMiD)	16 (76%)
Triple refractory	6 (29%)
Number of patients who received ASCT prior to VD‐PACE	18 (86%)

Among the 11 (52%) who had high‐risk cytogenetics, the ORR was 82% (*n* = 9): VGPR in 45%, PR in 36%, and stable disease in 9% of patients.

### 4.3 Survival

**FIGURE 1:**
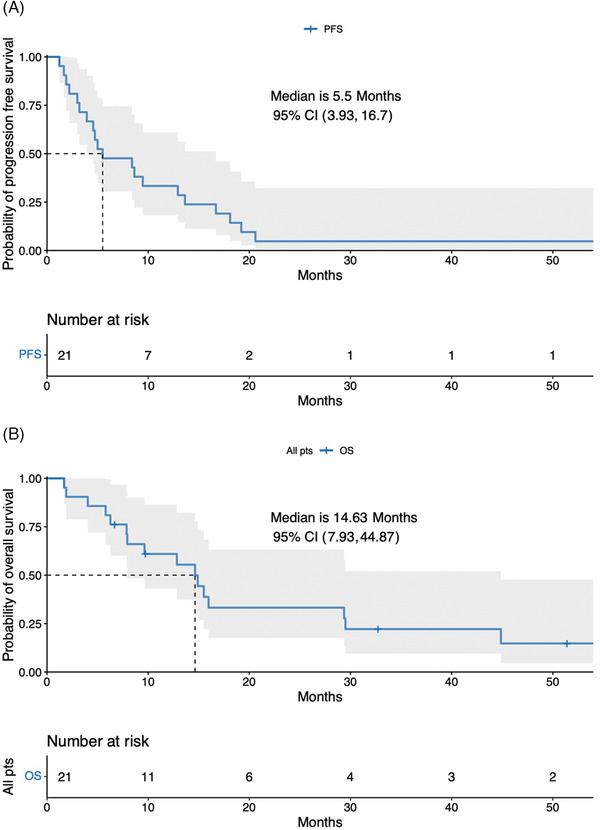
Kaplan Meier estimates of progression free survival for all patients (Panel A). Kaplan Meier estimates of overall survival for all patients (Panel B)

**FIGURE 2:**
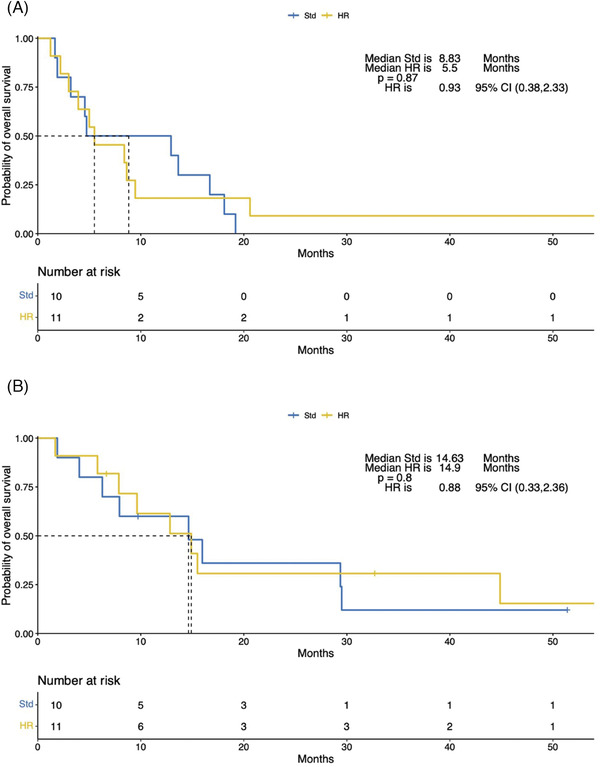
Kaplan Meier estimates of progression free survival for standard risk (Std) cytogenetics (Blue) and High risk (HR) cytogenetics (Yellow) (Panel A). Kaplan Meier estimates of overalls survival for Std cytogenetics (Blue) and HR cytogenetics (Yellow) (Panel B)

The median follow‐up for all patients was 51.4 months (range, 32.7–NR), the median progression‐free survival (PFS) was 5.5 months (95% CI: 3.93‐16.7) (Figure 1; panel A), and median OS was 14.6 months (95% CI: 7.93‐44.87) (Figure 1; panel B). Among the 11 patients (52%) with high‐risk cytogenetics EMD‐RRMM, the median PFS and overall survival (OS) were 5.5 months (95% CI: 3.9‐NA) and 14.9 months (95% CI: 7.8‐NA), respectively. High risk cytogenetics (HR) for PFS is 0.93 (95% CI: 0.38‐2.33) (Figure 2; panel A) and HR for OS is 0.88 (95% CI: 0.33‐2.36) (Figure 2; panel B).

Patients who underwent SCT had a median PFS and OS, measured from VDPACE + IMiD, of 15.5 months (95% CI: 11.3‐NA) and 26.8 months (95% CI: 12.3‐NA), respectively. Those who did not bridge to transplant after having achieved a response to VDPACE (*n* = 6) had a short median PFS of 3.6 months (95% CI: 2.2‐NA) and OS of 7.9 months (95% CI: 5.8‐NR).

**TABLE 4:** Response rate for VDPACE + IMiD

Response category	Relapsed/relapsed refractory myeloma number (%)
Overall response	16 (76%)
Complete response	2 (10%)
Very good partial response	7 (33%)
Partial response	7 (33%)
Minimal response	0
Stable disease	2 (10%)
Progressive disease	3 (14%)

Out of nine patients with CNS disease, five patients received a SCT post‐VDPACE‐based treatment with a median PFS of 15 months (95% CI:11.3‐NA) as measured from starting VDPACEtherapy.

### 4.4 Adverse events and related morbidity

All patients (*n*  = 21) were hospitalized during VDPACE administration for a minimum of 4 days. Observed toxicities included neutropenia (100%), anemia (95%), thrombocytopenia (100%), renal insufficiency (14%), neutropenic fever (48%), elevated liver function test (14%), venous thromboembolism (4%), mucositis (19%), vomiting (10%), gastrointestinal bleeding (10%), tumor lysis syndrome (14%), subdural hematoma (4%), and neuropathy (4%). The most common (>5%) grade 3/4 treatment‐related adverse events were neutropenia (100%), thrombocytopenia (100%), anemia (100%), febrile neutropenia (48%), gastrointestinal bleeding (10%), and tumor lysis syndrome (14%). Neutropenia and febrile neutropenia were managed by providing growth factors, including filgrastim and pegfilgrastim. The majority of grade 3/4 neutropenic events occurred within 3 weeks of the first dose. The median (range) duration of hospitalization for the treatment was 6 (4–30) days. Nine patients (43%) had readmission after administering chemotherapy, with a median duration of 4 (4–30) days during readmission. The most common cause of readmission was neutropenic fever.

Among 21 patients for whom the data were available, the median (range) number of platelet and red cell transfusions was 5 (0–15) units and 4 (0–15) units, respectively. At the end of the salvage treatment, all patients were transfusion independent, with no treatment‐related mortality (TRM).

## 5 DISCUSSION

Relapsed/refractory EMD RRMM is highly aggressive with a dismal prognosis. The treatment goals of relapsed myeloma have changed, emphasizing moving from long‐term disease‐free survival towards disease control and maintaining the quality of life. Treatment decisions at relapse depend on many factors, including performance status—morbidities, disease characteristics—such as cytogenetics and aggressiveness of the relapse, and characteristics of the previous response such as duration of response and associated drug toxicities. At the time of relapse, previous therapy lines and the duration of response should be considered [6].

Treatment options for MM have increased in recent years, and the introduction of novel PI and immunomodulatory drugs are significantly associated with prolonged survival in patients with MM. However, outcomes remain poor for patients with double refractory MM to both PI and IMiD drugs with an estimated 9–13 months survival [28].

In our analysis of 21 patients with EMD RRMM who received VDPACE + IMiD showed an ORR of 76% and a clinical benefit rate of 87%. Even in patients with triple refractory disease, the VDPACE +IMiD–based regimen maintained its efficacy. The VDPACE +IMiD regimen performed in EMD similarly to previously published experience in RRMM disease without EMD, with results also similar to other multichemotherapy regimens like DCEP (dexamethasone, cyclophosphamide, etoposide, and cisplatin) [29, 30, 31, 32, 33].

Despite impressive response rates, those responses were not deep or durable when compared with non‐EMD MM or standard‐risk MM [34]. However, patients with high‐risk cytogenetics performed similarly to patients with standard‐risk cytogenetics with regard to response rates. This similarity in response underscores the shortcoming of high‐risk MM definition based on cytogenetics alone because all patients with EMD, even those with standard cytogenetics, should be regarded as high‐risk. Similarly, previous publications showed that cytogenetics did not change the depth of response for 236 patients with RRMM treated with thalidomide and dexamethasone plus PACE [35]. These data suggest that this regimen is powerful in controlling the disease, albeit for a short time in some cases with EMD. However, it is hard to conclude from this response durability in EMD due to the low sample size.

In our study, ASCT followed VDPACE+IMiD treatment to improve the durability of responses. Those patients who underwent SCT had a median PFS and OS from VDPACE + IMiD of 15.5 and 26.8 months, respectively. Furthermore, the median time to first response was 2 months, but many patients achieved their best response beyond 2 months.

This retrospective single‐center study showed that VDAPACE/IMiD treatment strategy is feasible, with favorable tolerability and no TRM. VDAPACE/IMiD during salvage therapy was associated with predictable hematological toxicities. The absence of any TRM further suggests a favorable benefit‐to‐risk ratio. The major limitations of our analysis include our single‐center retrospective study design with associated limitations in data size, retrieval, interpretation, and absence of any comparator arm.

The safety profile of VDPACE + IMiD primarily consisted of hematologic adverse events (AEs), consistent with previous results. Despite cytopenias being common, the incidence of significant bleeding events or infections was low. Hematologic AEs were generally reversible and clinically manageable with dose delays, growth factor use, platelet transfusions, and appropriate supportive care. Nonhematologic grade 3/4 AEs were infrequent, with infections being the most common. Moreover, the frequency of infections was generally consistent with the expected infection rates in heavily pretreated patients.

In conclusion, our single‐institution experience demonstrates that patients with EMD can achieve a significant response rate using VDPACE+IMiD. Our data demonstrate that VDPACE+IMiD can be utilized as salvage therapy to control EMD MM and serves as a bridge for other treatments.

## FUNDING

There was no external funding for this study.

## CONFLICTS OF INTEREST

Dr. Ganguly reports the following conflicts: Seattle Genetics: Speakers Bureau; Daiichi Sankyo: Research Funding; Kite Pharma: Honoraria, Other: Advisory Board; Janssen: Honoraria, Other: Advisory Board.

Dr. McGuirk reports the following conflicts: Novartis: Research Funding; Fresenius Biotech: Research Funding; Astellas: Research Funding; Bellicum Pharmaceuticals: Research Funding; Kite Pharmaceuticals: Honoraria, Membership on an entity's Board of Directors or advisory committees, Research Funding, Speakers Bureau; Gamida Cell: Research Funding; Pluristem Ltd: Research Funding; Articulate Science LLC: Other: assistance with manuscript preparation; Juno Therapeutics: Honoraria, Membership on an entity's Board of Directors or advisory committees, Research Funding.

Dr Mahmoudjafari reports the following conflicts: Omeros: Advisory Board; Incyte: Advisory Board.

SA report honorarium from Celgene, Jansen, Karyopharm, GSK, Sanofi. Speakers Bureau: Celgene, Jansen, Sanofi.

## ETHICS APPROVAL

This retrospective chart review study involving human participants was in accordance with the ethical standards of the institutional and national research committee and with the 1964 Helsinki Declaration and its later amendments or comparable ethical standards. The Human Investigation Committee (IRB) of University of Kansas Medical Center approved this study. Consent to participate: This is a retrospective study, and no patients were formally consented.

## AVAILABILITY OF DATA AND MATERIAL

The data sources from which the results of this study were generated can be shared if requested.

## AUTHORS' CONTRIBUTIONS

Dr. Abdallah conceived the study idea and assisted in writing up the manuscript. Dr. Mahmoudjafari collected and analyzed the data and assisted in writing up the manuscript. Dr. Shune, Dr. Mohan, Dr. Cui, Dr. McGuirk, and Dr. Ganguly reviewed the final version of the manuscript and assisted in the critical review of the manuscript and data. SA reviewed, performed survival analysis, and assisted in writing the manuscript. All authors approved the final version of the manuscript and agree to be accountable for the integrity of all aspects of this work.
